# Reassessment of the risk of birth defects due to Zika virus in Guadeloupe, 2016

**DOI:** 10.1371/journal.pntd.0009048

**Published:** 2021-03-03

**Authors:** Anna L. Funk, Bruno Hoen, Ingrid Vingdassalom, Catherine Ryan, Philippe Kadhel, Kinda Schepers, Stanie Gaete, Benoit Tressières, Arnaud Fontanet

**Affiliations:** 1 Emerging Disease Epidemiology Unit, Institut Pasteur, Paris, France; 2 Sorbonne Université, Paris, France; 3 INSERM Centre d’Investigation Clinique 1424, Centre Hospitalier Universitaire de la Guadeloupe, Pointe-à-Pitre, France; 4 Centre Pluridisciplinaire de Diagnostic Prénatal, Centre Hospitalier Universitaire de la Guadeloupe, Pointe-à-Pitre, France; 5 Université des Antilles, Centre Hospitalier Universitaire de la Guadeloupe, Pointe-à-Pitre, France; 6 Institut de Recherche en Santé, Environnement et Travail (IRSET), Université de Rennes, Rennes, France; 7 Infectious Diseases Department, Centre Hospitalier Universitaire de la Guadeloupe, Pointe-à-Pitre, France; 8 Centre de Ressources Biologiques Karubiotec, Centre Hospitalier Universitaire de la Guadeloupe, Pointe-à-Pitre, France; 9 Unité Pasteur-CNAM Risques Infectieux et Émergents, Conservatoire National des Arts et Métiers, Paris, France; University of Glasgow, UNITED KINGDOM

## Abstract

**Background:**

In the French Territories in the Americas (FTA), the risk of birth defects possibly associated with Zika virus (ZIKV) infection was 7.0% (95%CI: 5.0 to 9.5) among foetuses/infants of 546 women with symptomatic RT-PCR confirmed ZIKV infection during pregnancy. Many of these defects were isolated measurement-based microcephaly (i.e. without any detected brain or clinical abnormalities) or mild neurological conditions. We wanted to estimate the proportion of such minor findings among live births of women who were pregnant in the same region during the outbreak period but who were not infected with ZIKV.

**Methods:**

In Guadeloupe, pregnant women were recruited at the time of delivery and tested for ZIKV infection. The outcomes of live born infants of ZIKV non-infected women were compared to those of ZIKV-exposed live born infants in Guadeloupe, extracted from the FTA prospective cohort.

**Results:**

Of 490 live born infants without exposure to ZIKV, 42 infants (8.6%, 95%CI: 6.2–11.4) had mild abnormalities that have been described as ‘potentially linked to ZIKV infection’; all but one of these was isolated measurement-based microcephaly. Among the 241 live born infants with ZIKV exposure, the proportion of such abnormalities, using the same definition, was similar (6.6%, 95%CI: 3.8–10.6).

**Conclusions:**

Isolated anthropometric abnormalities and mild neurological conditions were as prevalent among infants with and without in-utero ZIKV exposure. If such abnormalities had not been considered as ‘potentially linked to ZIKV’ in the original prospective cohort in Guadeloupe, the overall estimate of the risk of birth defects considered due to the virus would have been significantly lower, at approximately 1.6% (95% CI: 0.4–4.1).

**Trial registration:**

ClinicalTrials.gov (NCT02916732)

## Author summary

Microcephaly defined using growth charts occurs naturally in some healthy infants. Nonetheless, in many recent Zika virus studies, such measurement-based microcephaly and other common mild abnormalities have been automatically attributed to viral exposure during pregnancy. For example, in 2016 in Guadeloupe, we did a cohort study and estimated that the risk of Zika virus related birth defects was 7%. However, most of the ‘abnormal’ live born infants that contributed to this risk estimate were considered healthy by pediatricians, but were found to have measurement-based microcephaly during data analysis. In the current study, we did a cross-sectional evaluation of infants live born in 2016 in Guadeloupe, but whose mothers were not infected with Zika virus during pregnancy. We found that Zika virus unexposed live born infants had similar proportions of measurement-based microcephaly and mild neurological abnormalities as exposed live born infants. If only severe and uncommon birth defects were considered linked to Zika virus in our original cohort study, our risk estimate would have been significantly lower. This research shows that it is important to consider baseline risks and control comparisons when establishing key parameters for emerging diseases, such as Zika virus.

## Introduction

Since the first evidence surfaced that linked Zika virus (ZIKV) to fetal microcephaly and other brain abnormalities,[1,2] key research priorities have been to define the range of defects associated with ZIKV infection during pregnancy, as well as to establish the risk of a fetus or infant being affected following ZIKV infection during pregnancy. A handful of case-series and case-control studies have now been summarized to establish a preliminary definition of Zika congenital syndrome (ZCS), which includes a range of ocular abnormalities and neurological defects, such as microcephaly, structural brain abnormalities, consequences of central nervous system dysfunction, swallowing disorders, irritability, seizures, neurodevelopmental issues, and others.[3–9] In terms of the overall risk of abnormalities following maternal ZIKV infection during pregnancy, this has been estimated as 46% (95%CI: 37–56) in a prospective cohort study from Brazil using a broader definition for ZIKV-associated outcomes, 5% (95%CI: 4–6) in a registry-based study in the United States of America, and 7% (95%CI: 5–10) and 13% (95%CI: 9–18) through two separate prospective cohort studies in the French Territories in the Americas.[10–13]

For the purpose of determining those birth defects that can actually be linked to ZIKV in an exposed population, an estimation of the baseline level of birth defects in an appropriate ZIKV non-infected control group is necessary. There has been minimal use of comparative control groups within prospective studies on this topic.[10,14,15] In Brazil, a prospectively followed control group demonstrated that total adverse outcomes were significantly fewer in women without evidence of ZIKV: 11% (95%CI 5–22) versus 46% (95%CI: 37–56); however, for some specific outcome categories, such as fetal demise and infants with microcephaly, there was no difference.[10] In Guadeloupe, in the absence of a prospective cohort of pregnant women without ZIKV infection, we recruited, at delivery, women and their live born infants who were known to have not been infected with ZIKV. We compared the proportion of congenital abnormalities in this ZIKV-negative group with that observed among a subset of live born infants (ie. all of those from Guadeloupe) from our prospective cohort of women in the French Territories in the Americas who developed symptomatic RT-PCR confirmed ZIKV infection at any time during pregnancy.[12]

## Methods

### Ethics statement

The ZIKA-DFA-FE study is registered with ClinicalTrials.gov (NCT02916732) and received ethics approval by the Comité de Protection des Personnes Sud-Ouest et Outremer III (CEBH2016/03), in compliance with the Declaration of Helsinki. All women provided written informed consent to have their, and their infants’, data included in this study.

### Background information

The ZIKA-DFA-FE cohort study (an acronym for “Zika in the French Territories in the Americas in Pregnant Women”), which has been described elsewhere,[12] used four different recruitment methods in an attempt to observe women whose pregnancies overlapped with the 2016 ZIKV epidemic period in the French Territories in the Americas (Guadeloupe, Martinique, French Guyana): 1) pregnant women with symptoms consistent with ZIKV infection, 2) pregnant women for whom a fetal abnormality was detected during routine fetal ultrasound examinations, 3) pregnant women who experienced fetal demise, and 4) pregnant women who delivered at participating hospitals during or up to nine months following the ZIKV epidemic period. A prospective cohort of women infected with ZIKV during pregnancy was derived from the first recruitment method,[12] and a cross-sectional study of women and their live born infants not infected with ZIKV during pregnancy was derived from the fourth recruitment method, which will be described below.

### Data collection

At the time of hospital admission for labor, each eligible woman was informed of the study and invited to participate; oral consent was obtained before delivery and written informed consent was obtained before delivery whenever possible or within 24 hours after delivery otherwise. A questionnaire exploring socio-demographic characteristics, such as age, ethnicity, education, profession, and lifestyle factors, was administered. Clinical information, including the number of previous pregnancies, history of adverse pregnancy outcomes, medical history, clinical symptoms consistent with ZIKV infection, and any other clinically significant medical event during pregnancy, was also collected. Exactly as within the prospective cohort study, infant clinical data such as gestational age, length, weight, and head circumference, APGAR scores (1, 5 and 10 minutes of life) were collected on the day of birth, and a standardized clinical examination was carried out in the first four days of life. The medical files of participants were reviewed to collect data on clinical and ultrasound examinations that were performed during the pregnancy.

### Laboratory testing

All participating women had blood sampled on the day of delivery, from which sera were frozen and stored. These were tested for the presence of anti-ZIKV IgG antibodies, using the Euroimmun ZIKV IgG immunoassay (Euroimmun, Medizinische Labordiagnostika AG, Lübeck, Germany). The results of any other ZIKV tests (serological or molecular) or TORCH testing that had been performed on biological samples collected during the pregnancy were also recorded.

### Study participants

For the cross-sectional study, we selected women from the fourth recruitment method of the ZIKA-DFA-FE study who gave birth to a live neonate during or up to 9 months following the 2016 ZIKV epidemic period (in Guadeloupe, the epidemic lasted from April 4^th^ to September 19^th^ 2016), and who had a confirmed negative IgG serology test for ZIKV from blood taken at the time of delivery as well as no other positive or indeterminate ZIKV test during pregnancy.

Anthropometric abnormalities and other birth defects in infants were classified according to the same case definitions as used in the prospective, ZIKV-exposed cohort study.[12] Microcephaly was defined as moderate when head circumference was between– 2 SD and– 3 SD and severe when head circumference was less than– 3 SD, based on the INTERGROWTH-21^st^ standards (http://intergrowth21.ndog.ox.ac.uk/) for gestational age and sex. Moderate microcephaly was further defined as proportionate or disproportionate depending on whether the neonate was small for gestational age (ie. birth weight less than -1.28 SD).[10,12,16] Clinical examination records and ultrasound files of participants were reviewed for evidence of birth defects that are considered to be potentially associated with ZIKV infection according to the current definition of ZCS, including: structural brain abnormalities (e.g. calcifications, ventriculomegaly, lissencephaly), eye abnormalities, hearing impairment, and other consequences of central nervous system dysfunction (e.g arthrogryposis, clubfoot).[6,12]

### Statistical analysis

We compared infant outcomes from the prospective cohort study of women infected with ZIKV during pregnancy,[12] with those from the cross-sectional study of women not infected with ZIKV during pregnancy. For both of these ZIKA-DFA-FE study populations only the subset of women and infants from Guadeloupe, were considered. Only outcomes of live born infants were compared due to the unavailability of data on fetal demise in ZIKV unexposed pregnancies where recruitment took place at the time of delivery. Baseline characteristics of women were compared using the Student’s t test for continuous variables and chi-square or Fisher’s exact test for categorical variables. The proportion of live born infants with anthropometric abnormalities and other defects was compared using Fisher’s exact test. The odds ratio (OR) for the association between abnormalities in live born infants and Zika virus exposure during pregnancy was estimated through a multiple logistic regression model that included maternal age, occupational category, and ethnicity. Maternal age was categorized as 18–24, 25–34, 35–39, or 40+ years. Three groups of occupational categories were used: unemployed and student participants; employees and labourers; business owners, highly qualified professionals and intermediate professions (e.g. teachers, nurses). Ethnicity was self-reported by mothers and categorized as follows: any sub-Saharan African or Amerindian ancestry (including those indicating ancestry as ‘Guadeloupe’, ‘Antilles’, or similar), other ancestry (e.g. Indian, North African, South East Asian) without sub-Saharan African or Amerindian ancestry, European ancestry only. Data were analyzed by ALF using Stata 13.1 (StataCorp LP Lakeway, TX, USA).

## Results

### Participants

**Fig 1:**
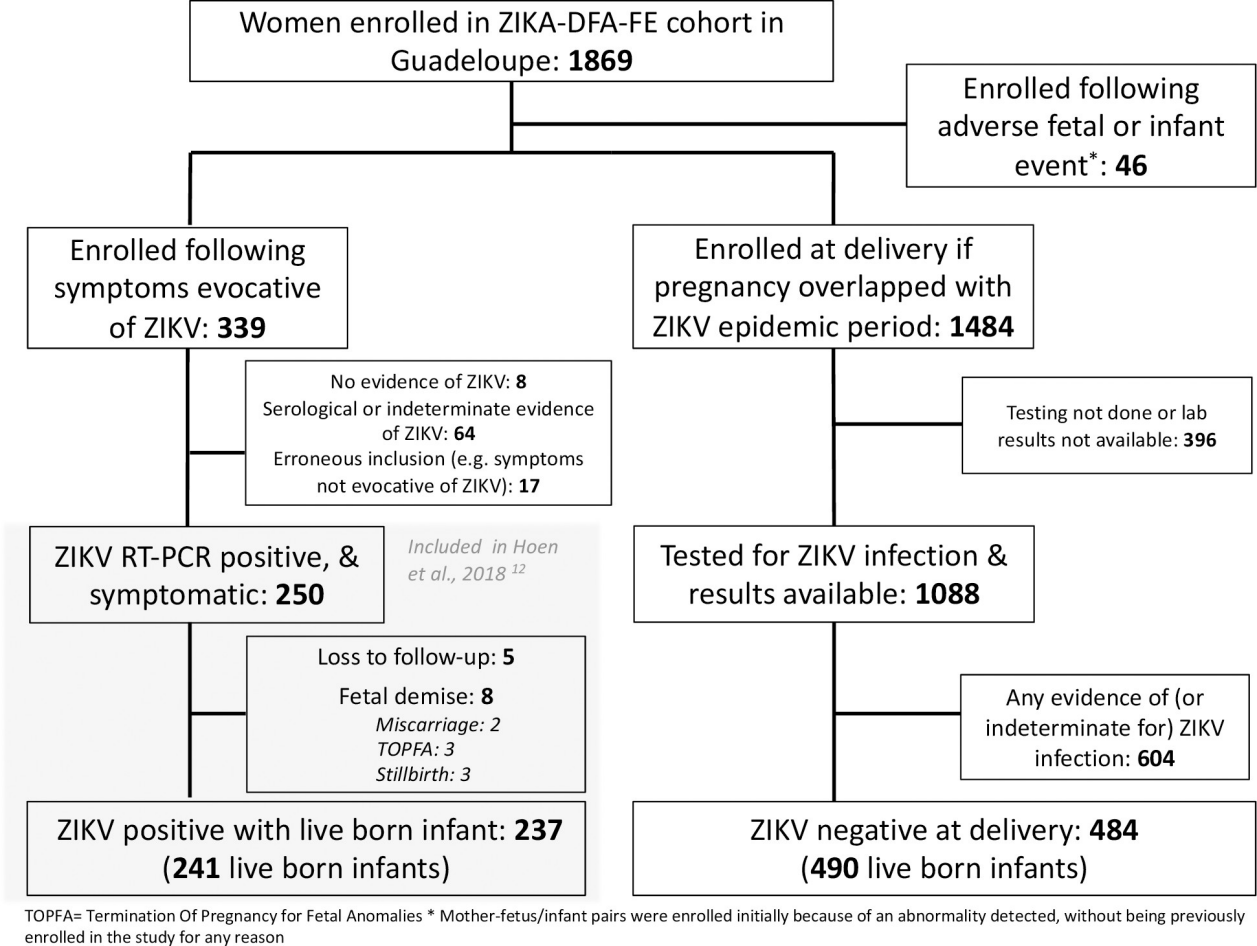
Flow chart of inclusion of ZIKV un-exposed and exposed pregnant women for inclusion of live births in this analysis, Guadeloupe, 2016.

Of the 1484 women enrolled in the cross-sectional study in Guadeloupe, 484 had a negative IgG serology test for ZIKV at the time of delivery. Of these women, 395 additionally had ZIKV IgM testing, and one woman had ZIKV PCR testing, on the blood sample taken at the time of delivery—all results were negative. Of these 484 women, 6 had twin pregnancies and therefore the outcomes of 490 live born infants were observed. In the prospective cohort, of the 250 pregnant women in Guadeloupe who had symptomatic, RT-PCR confirmed ZIKV infection at any time during pregnancy, 245 pregnancies were followed up until the time of the pregnancy outcome. Within this group there were eight cases of fetal demise and four sets of twins, and therefore the outcomes of 241 ZIKV exposed live born infants were observed. *See Fig 1.*

The mean age of ZIKV non-infected women was 30.7 years (SD = 6.4), and that of ZIKV infected women was 30.0 years (SD = 6.3). There was a higher proportion of reported smoking in the ZIKV-infected cohort during pregnancy compared to the ZIKV non-infected: 4.2% (95%CI: 2.0–7.6%) versus 0.6% (95%CI: 0.4–1.8%). There were also more unemployed women in the ZIKV non-infected group compared to the ZIKV-infected one: 48.1% (95%CI: 43.3–52.4) versus 34.6% (95%CI: 28.6–41.0). *See Table 1*.

**Table 1:** Baseline characteristics of ZIKV non-infected and infected^12^ women from Guadeloupe who delivered live born infants, 2016.

Characteristic	ZIKV non-infected (N = 484)	ZIKV infected (N = 237)
**Age—yr (mean and range)**	30.7 (18–46)	30.0 (18–46)
**Age category—no. (%)**		
18–24 years	92 (19.0)	50 (21.1)
25–34 years	237 (49.0)	123 (51.9)
35–39 years	113 (23.4)	47 (19.8)
40 + years	42 (8.7)	17 (7.2)
*Missing*	0	0
**Occupation—no. (%)**		
Student	13 (2.7)	6 (2.5)
Unemployed	233 (48.1)*	82 (34.6)*
Employee	148 (30.4)	91 (38.4)
Laborer	1 (0.2)	1 (0.4)
Business owners	14 (2.9)	17 (7.2)
Highly qualified or qualified professional	75 (15.5)	39 (16.5)
*Missing data or declined to respond*	0	1 (0.4)
Ethnicity^**^—no. (%)		
Any sub-Saharan African or Amerindian ancestry	344 (71.1)	163 (68.8)
Other ancestry (e.g. Indian, North African, East Asian)	77 (15.9)	25 (10.6)
European ancestry only	62 (12.8)	41 (17.3)
*Missing data or declined to respond*	1 (0.2)	8 (3.4)
**Medical history—no. (%)**		
Arterial hypertension	12 (2.5)	7 (3.0)
Diabetes	12 (2.5)	4 (1.7)
Sickle cell disease	7 (1.5)	2 (0.8)
**Previous pregnancies—no. (%)**		
0	110 (22.7)	65 (27.4)
1	133 (27.5)	57 (24.1)
2	109 (22.5)	52 (21.9)
> = 3	130 (26.9)	63 (26.6)
*Missing*	2 (0.4)	0
**Previous adverse pregnancy outcomes—no. (%)**		
Congenital abnormalities	2 (0.4)	3 (1.3)
Stillbirth	6 (1.2)	2 (0.8)
Termination of pregnancy for medical reasons	4 (0.8)	4 (1.7)
**Lifestyle practices during this pregnancy—no. (%)**		
Alcohol consumption	0	0
Drug use	1 (0.2)	2 (0.8)
Current smoker	3 (0.6)*	10 (4.2)*

* Comparison between Zika non-infected and infected women with p = 0.001

### Anthropometric abnormalities and other birth defects in live born infants

Of the 490 live born infants of women without ZIKV infection during pregnancy, 66 (13.5%, 95%CI: 10.6–16.8) were small for gestational age, and 41 (8.4%, 95%CI: 6.1–11.2) had microcephaly. One of these cases of moderate microcephaly had a possible genetic aetiology (Adams Oliver syndrome). None of the 41 cases of microcephaly were identified as such by the attending clinicians (i.e. they were defined later after comparing infant measurements to a standardized growth curve) and for none were there reports of other structural brain or clinical abnormalities. Additionally, one infant (0.2%, 95%CI: 0.01–1.1) had an abnormality that could be a consequence of CNS dysfunction, which was clubfoot. Five infants (1.0%, 95%CI: 0.3–2.4) had other abnormalities that are not considered as linked to ZIKV infection, including: supplementary fingers or toes (n = 2), anal imperforation (n = 1), urinary tract abnormalities (n = 2). Besides two infants born to HIV-infected mothers who were small-for-gestational-age, there were no other abnormalities identified in the remaining four infants born to mothers with TORCH infections. *See Table 2*.

**Table 2:** TORCH results in ZIKV non-infected and ZIKV-infected [12] women giving birth in Guadeloupe during or up to 9 months after the 2016 ZIKV epidemic period (2016–2017).

	ZIKV non-infected (N = 484)	ZIKV infected (N = 237)
**Positive results on any TORCH test**	**6** (1.2)	**5** (2.1)
**Toxoplasmosis**		
Tested	468 (96.9)	219 (92.4)
Positive	3 (1.0)	0
**Syphilis**		
Tested	249 (51.6)	184 (77.6)
Positive	0	2 (0.8)
**HIV**		
Tested	449 (93.0)	188 (79.3)
Positive	3 (0.6)	2 (0.8)
**Rubella**		
Tested	464 (96.1)	199 (84.0)
Positive	0	0
**Cytomegalovirus**		
Tested	17 (3.5)	36 (15.2)
Positive	0	1 (0.4)

There was no significant difference in risk of anthropometric or other mild birth defects in live born infants whose mothers had a symptomatic PCR-confirmed ZIKV infection during pregnancy compared to those whose mothers had no evidence of prior ZIKV infection at the time of delivery in Guadeloupe (6.6%, 95%CI: 3.8–10.6, versus 8.6%, 95%CI: 6.2–11.4, respectively). *See Table 3.*

**Table 3:** Abnormalities in *live births* of ZIKV non-infected and infected [12] women in Guadeloupe during or up to 9 months after the 2016 ZIKV epidemic period.

	ZIKV non-infected (N = 490)	ZIKV infected (N = 241)
**Any neurological or ocular abnormalities**	**42 (8**.**6)**	**16 (6**.**6)**
Microcephaly (<-2SD)	41 (8.4)	12 (5.0)
*Severe microcephaly alone*	11 (2.2)	1 (0.4)
*Moderate-disproportionate alone*	10 (2.0)	6 (2.5)
*Moderate-proportionate alone*	19 (3.9)	4 (1.7)
*Severe or moderate microcephaly with other neurological abnormalities*	0	0
*Severe or moderate microcephaly with a genetic or chromosomal syndrome*	1 (0.2)	1 (0.4)
*Missing*	6 (1.2)	5 (2.1)
Structural brain abnormalities	0	1 (0.4)
Ocular abnormalities	0	0
Neural tube defects	0	1 (0.4)
Consequences of CNS dysfunction	1 (0.2)	2 (0.8)^^^
**Other abnormalities**	**5 (1**.**0)**	**2 (0**.**8)**
Skeletal abnormalities	2 (0.4)	2 (0.8)^^^
Other	3 (0.6)	0
**Small for gestational age (weight <-1.28 SD)** *(with or without any of the above abnormalities)*	**66 (13**.**5)**	**33 (13**.**7)**
*Missing*	*1 (0*.*2)*	*3 (1*.*2)*

**^** One infant represented in each category as they had both club-foot and polydactyly**. Note:** No significantly different values

There was no association (OR 0.8, 95%CI: 0.4–1.4) between ZIKV exposure and abnormalities in live born infants in a multiple logistic regression model adjusting for age, occupation, and ethnicity. When compared to women 25–34 years of age, women 18–24, 35–39, and 40 or more years of age were more likely to have a live born infant with abnormalities with ORs of 5.1 (95%CI: 2.5–10.7), 2.3 (95% CI: 1.0–5.0) and 2.9 (95% CI: 1.0–8.0), respectively. There was no association between either occupation or ethnicity and the likelihood of having a live born infant with abnormalities. *See Table 4.*

**Table 4:** Association of Zika virus exposure during pregnancy and other maternal characteristics with abnormalities in 731 live born infants.

	Odds Ratio	95% CI
**Zika virus exposure during pregnancy**	0.8	0.4–1.5
**Maternal age**		
18–24 years	5.1	2.5–10.7
25–34 years	Reference	-
35–39 years	2.3	1.0–5.0
40 + years	2.9	1.0–8.0
**Occupational category**		
Unemployed or student	Reference	-
Employee or laborer	1.3	0.7–2.5
Business owner or qualified professional	0.6	0.2–1.7
**Ethnicity**		
Any sub-Saharan African or Amerindian ancestry	Reference	-
Other ancestry (e.g. Indian, North African, East Asian)	0.4	0.1–1.1
European ancestry only	0.6	0.2–1.7

Of the 16 abnormalities seen among 241 live born infants of women with ZIKV infection during pregnancy (*Table 3*), 14 of these were either isolated anthropometric abnormalities (i.e. measurement-based microcephaly) or isolated mild CNS dysfunction defects (i.e. clubfoot), as well as one case of spina bifida (no longer considered as linked to ZIKV infection[17,18]), and one case of ventriculomegaly.[12] In the original prospective cohort, there were an additional eight pregnancies with known outcomes from Guadeloupe (i.e. a total of 249 infants/fetuses), all of which led to fetal demise and were not included in the current comparative analysis of live born infants (*See Fig 1*). Of these eight cases of fetal demise, there were two instance of miscarriage and three instances of stillbirth without any evidence of fetal anomalies. The other three instances of fetal demise were terminations of pregnancy for fetal anomalies (TOPFA) that were severe and suggestive of Zika Congenital Syndrome. In our original report, all neurological or ocular birth defects from Guadeloupe that were potentially linked to ZIKV infection in Guadeloupe were included in our risk estimate and so this included the 16 abnormalities in live born infants as well as the three cases of TOPFA (n = 19/249; 7.6%, 95%CI: 4.7–11.7).[12] If, as may be justified by the current study, isolated anthropometric abnormalities and other mild birth defects in live born infants had not been definitely considered as ‘potentially linked to ZIKV’ in the original ZIKV-exposed prospective cohort study, then the number of ZIKV-related abnormalities in live born infants would have been counted as one (i.e. one case of ventriculomegaly). This one case, combined with the severe neurological abnormalities in the three cases of TOPFA, brings our estimate down to 1.6% (95%CI: 0.4–4.1%). This would have translated to a 4.1% (95%CI: 0.9–11.5%), 0.8% (95%CI: 0.02–4.6%), and 0% (one-sided 97.5%CI:0–6.3%) risk of birth defects per first, second, and third trimester, respectively.

## Discussion

We found no statistically significant difference in the risk of neurological birth defects in live born infants of ZIKV infected and non-infected women in Guadeloupe during the 2016 epidemic period. This can primarily be explained by the fact that most of the abnormalities reported as ‘potentially linked to ZIKV’, for both the exposed and non-exposed pregnancies in this study, represent identification of isolated microcephaly in live births; these cases were defined based only on anthropometric measurements, with known clinical and radiological findings for each infant being normal.[12] This diagnostic approach to microcephaly, which does not require clinical judgment on the appearance of microcephaly, but relies solely on the comparison of a head circumference measurement against a normalized birth curve, has been used in all of the prospective cohort studies describing the risk of birth defects following maternal ZIKV exposure during pregnancy, to date.[10–15] However, defining microcephaly based on ‘metrics’ does not reflect the real-life clinical diagnosis of this condition, and can lead to a false surge in cases if applied to an entire population for surveillance purposes.[19] Registries using more stringent definitions (e.g. -3SD) and/or clinician specific criteria indicate that true disease-related microcephaly is very rare; the European Surveillance of Congenital Anomalies (EUROCAT) recently estimated the prevalence of microcephaly in Europe to be 1.53 per 10,000 births (~0.02%) with data from 2012–2016.[20] As infant growth is approximately normally distributed, the INTERGROWTH-21^st^ study itself prescribes that approximately 2% and 0.1% of healthy infants should have a head circumference at birth that falls below -2 and -3SD, respectively, on their pooled international growth standard.[16] The INTERGROWTH-21^st^ study noted varying levels of fit for individual populations when compared to their pooled standard,[16] which could exacerbate the proportion of otherwise normal infants falling below these thresholds.

This is the largest study of ZIKV non-infected women from a defined epidemic region that has been used as a comparative control group against ZIKV-infected pregnant women followed up during pregnancy. It was conducted in a resource-rich setting where the standard of care during pregnancy is high. The exposure statuses of each of the two groups included were well defined. The ZIKV infected women from Guadeloupe were confirmed via RT-PCR within days of infection.[12] Relying solely on PCR testing for defining the ZIKV-exposed group avoided difficulties in interpreting serological results in a region where other flaviviruses co-circulate. The ZIKV non-infected group was defined so based on the absence of ZIKV IgG at the time of delivery. ZIKV IgG appears rapidly after infection and remains detectable for a follow-up of four or more months.[21,22] Several studies have demonstrated 100% sensitivity of the ZIKV IgG Euroimmun assay for detecting antibodies soon after infection and for several months, including: 124 ZIKV-infected individuals in French Guiana sampled between 30 and 180 days after symptom onset;[23] 65 pregnant women from the ZIKA-DFA-FE cohort in Guadeloupe with RT-PCR confirmed ZIKV infection (14 women were infected in the first trimester) sampled from the time of symptom onset up until delivery;[24] and finally, from the manufacturer’s own evaluation.[25] A 100% sensitivity of ZIKV IgG testing at delivery to detect infection during pregnancy translates into a 100% negative predictive value, i.e., women with a negative test at delivery had not been infected during pregnancy.

This study has several limitations. As we had no directly comparable prospective cohort, we used a group of ZIKV non-infected women delivering at the same hospitals and in the same time period. Our aim was not to compare the risk of birth defects from conception to delivery in the two groups, since this information was not available for the ZIKV non-infected group, but to compare the proportion of microcephaly and other birth defects among live born infants, to see if the minor abnormalities found in the ZIKV-exposed group could be confidently related to ZIKV infection. If the ZIKV non-exposed group had been followed prospectively in a similar fashion to the ZIKV exposed pregnancies, this would have allowed for a more complete comparison of the risk of abnormalities and adverse outcomes. There is limited recent baseline data on the incidence of pregnancy loss in Guadeloupe, specifically. One 2004 study from centres across France, including Guadeloupe and Martinique, found an overall TOPFA incidence at any gestational age of 0.7%.[26] In 2005 in Paris, the incidence of TOPFA after 26 weeks gestation was estimated as 0.2%.[27] The Pan American Health Organization reported the incidence of stillbirth among women in Guadeloupe (2001–2003) to be 1.6%, with a decreasing trend.[28] The French Pregnancy Cohort found an incidence of 18.6% and 0.7% for miscarriage and stillbirth across France (2010–2013), respectively.[29] Of 249 fetuses/infants in our Zika virus exposed cohort, there were three instances each of stillbirth and TOPFA (each 1.2% (95%CI: 0.3–3.5), as well as two miscarriages (0.8% (95%CI: 0.1–2.9). Therefore, our incidence of stillbirth seems comparable to previous findings, whereas our incidence of TOPFA may be similar or slightly higher. Miscarriage incidence is artificially low in our Zika virus exposed cohort as participants were recruited at varying stages of pregnancy. The exposed and unexposed groups were similar in terms of the prevalence of TORCH infections as well as for most baseline characteristics; however, ZIKV non-infected women had more unemployment, and more ZIKV infected women reported smoking during pregnancy. This may reflect recall ability and employment situation differences according to the timing of data collection, as ZIKV non-infected women were all recruited at the time of delivery and ZIKV infected women were recruited at various earlier time points during their pregnancy. Furthermore, the quality of follow-up and collection of data on the pregnancy was higher in the prospective ZIKV-exposed symptomatic cohort. Highlighting this is the fact that ultrasound records were available for 88.4% of ZIKV infected women and only 51.6% for ZIKV non-infected women. However, such a difference would most likely lead to an underestimation of birth defects in the ZIKV non-infected group. Furthermore, the completeness of data at the time of delivery for live births, which was used to determine anthropometric and clinically apparent abnormalities, was very high in both ZIKV-exposed (97.9%) and non-exposed (98.8%) infants. It is also important to note that in the ZIKV-exposed infants in Guadeloupe, third trimester prenatal ultrasound results which could confirm a lack of underlying structural brain abnormalities, were not available for 3 of 11 infants (27%) who are identified as having ‘isolated microcephaly’.[12] The mothers of these infants were infected with ZIKV in the first trimester (n = 1), second trimester (n = 1), and third trimester (n = 1). If any of these infants had underlying structural brain abnormalities, such as calcifications, then our stated adjusted risk of birth defects linked to ZIKV would be an underestimate. A further limitation of our study is our inability to include the risk of some abnormalities that may be detected at a later stage of the infants’ lives, such as ocular defects. Only one prospective cohort study to date has given such an estimate: 6.0% in Brazil.[10] This risk estimate is unlikely to apply to live born infants in our setting; all infants with severe ocular defects (such as macular lesions) in the prospective Brazilian cohort had severe structural brain abnormalities, including microcephaly and calcifications, detected by ultrasound prenatally, and in our setting, all fetuses with severe structural brain abnormalities detected prenatally were medically aborted. One live born infant in our ZIKV-exposed group did have moderate structural brain abnormalities detected prenatally (i.e. ventriculomegaly), in this infant, an eye examination after birth showed no abnormalities. Finally, various effect modifiers, such as socioeconomic status and previous infection with other arboviruses, have been proposed to potentially increase the risk of Zika Congenital Syndrome when women are infected with ZIKV during pregnancy.[30–32] It would be reasonable to believe that such factors could also lead to an increased risk for mild and anthropometric abnormalities regardless of ZIKV infection. Although our multiple logistic regression did not find any association of abnormalities in live born infants with occupational category or ethnicity, other indicators of socioeconomic status (e.g., household income, education level) were unmeasured. Additionally, very little dengue virus testing was done. Therefore, this study is not able to explore the differences in prevalence of past or current dengue virus infection between the ZIKV-exposed or unexposed groups, or to determine the effect that this may have on the incidence of abnormalities in live born infants.

This study highlights the importance of a control group for establishing the baseline risk of anthropometric and other birth defects when determining the risk of congenital abnormalities that can be linked to a given infection during pregnancy. This is particularly true for abnormalities defined by anthropometric measurements, where regional variations may exist;[18,33–35] in these instances, the use of regionally specific growth standards as well as standardized international growth standards would be of interest. When a control group comparison is not possible, published baseline estimates from a pre-infection (e.g., pre-Zika virus) time period may be useful for contextualizing findings. However, the compatibility of the definitions used for birth defects in previous literature with those of any current study should be scrutinized prior to ‘before and after’ comparisons. For example, the Latin American Collaborative Study of Congenital Malformations, which relies on reporting of microcephaly to birth defects registries, determined a microcephaly incidence of 0.05% in Brazil between 2010 and 2014.[19] However, another study in two regions of Brazil in 2010 used a metric microcephaly definition with the INTERGROWTH-21^st^ standards and found incidences of 2.5% and 3.5%.[36]

In our case, reassessment of the risk of birth defects from a ZIKV exposed prospective cohort after consideration of mild abnormalities seen in a time and place-matched control group, indicates an initial significant overestimation of the risk of birth defects potentially associated with ZIKV at the time of birth. Our new estimates of approximately 4%, 1%, and 0% for the risk of birth defects following ZIKV infection in first, second, and third trimester, respectively, are comparable to those from a recent meta-analysis of prospective studies on this topic.[37] Still, as with other congenital infections that cause neurological abnormalities, such as cytomegalovirus and rubella,[38,39] longer term studies that postnatally follow-up infants exposed to ZIKV in-utero, but who are apparently healthy at birth, are needed in order to understand additional ocular, hearing, and developmental abnormalities and derive the true overall risk of defects. For these latter infant cohorts, consideration of the proportion of hearing and developmental abnormalities, which are non-invasive examinations, in a ZIKV non-exposed control group, through testing performed with the same vigilance, will continue to be of major importance.

## References

[1] Calvet G, Aguiar RS, Melo ASO, Sampaio SA, de Filippis I, Fabri A, et al. Detection and sequencing of Zika virus from amniotic fluid of fetuses with microcephaly in Brazil: a case study. Lancet Infect Dis 2016; 16(6):653–60. 10.1016/S1473-3099(16)00095-5 26897108

[2] Cordeiro MT, Pena LJ, Brito CA, Gil LH, Marques ET. Positive IgM for Zika virus in the cerebrospinal fluid of 30 neonates with microcephaly in Brazil. Lancet 2016; 387(10030): 1811–2. 10.1016/S0140-6736(16)30253-7 27103126

[3] de Araujo TVB, de Alencar Ximenes RA, de Barros Miranda-Filho D, Valongueiro S, de Albuquerque MFPM, Braga C, et al. Association between microcephaly, Zika virus infection, and other risk factors in Brazil: final report of a case-control study. Lancet Infect Dis 2018; 18(3): 328–36. 10.1016/S1473-3099(17)30727-2 29242091 PMC7617036

[4] Krow-Lucal ER, de Andrade MR, Cananéa JNA, Moore CA, Leite PL, Biggerstaff BJ, et al. Association and birth prevalence of microcephaly attributable to Zika virus infection among infants in Paraíba, Brazil, in 2015–16: a case-control study. Lancet Child Adolesc Health 2018; 2(3): 205–13 10.1016/S2352-4642(18)30020-8 30169255

[5] Santa Rita TH, Barra RB, Peixoto GP, Mesquita PG, Barra GB. Association between suspected Zika virus disease during pregnancy and giving birth to a newborn with congenital microcephaly: a matched case-control study. BMC Res Notes 2017; 10(1): 457. 10.1186/s13104-017-2796-1 28877754 PMC5588708

[6] Moore CA, Staples JE, Dobyns WB, Pessoa A, Ventura CV, da Fonseca EB, et al. Characterizing the pattern of anomalies in congenital Zika syndrome for pediatric clinicians. JAMA Pediatr 2017; 171(3): 288–95 10.1001/jamapediatrics.2016.3982 27812690 PMC5561417

[7] Dias JRO, Ventura CV, Freitas BP, Prazeres J, Ventura LO, Bravo-Filho V et al. Ocular abnormalities in congenital Zika syndrome: are the ophthalmoscopic findings "the top of the iceberg"? Prog Retin Eye Res 2018; 10.1016/j.preteyeres.2018.04.004 29698814

[8] Oliveira-Filho J, Felzemburgh R, Costa F, Nery N, Mattos A, Henriques DF, et al. Seizures as a Complication of Congenital Zika Syndrome in Early Infancy. Am J Trop Med Hyg 2018; 98(6): 1860–62 10.4269/ajtmh.17-1020 29692307 PMC6086187

[9] Wheeler AC. Development of Infants With Congenital Zika Syndrome: What Do We Know and What Can We Expect? Pediatrics 2018; 141(Suppl 2): S154–S60. 10.1542/peds.2017-2038D 29437048 PMC5795516

[10] Brasil P, Pereira JP, Moreira ME, Nogueira RMR, Damasceno L, Wakimoto M, et al. Zika Virus Infection in Pregnant Women in Rio de Janeiro. N Engl J Med 2016; 375(24):2321–34. 10.1056/NEJMoa1602412 26943629 PMC5323261

[11] Shapiro-Mendoza CK, Rice ME, Galang RR, Fulton AC, VanMaldeghem K, Prado MV, et al. Pregnancy Outcomes After Maternal Zika Virus Infection During Pregnancy—U.S. Territories, January 1, 2016-April 25, 2017. MMWR Morb Mortal Wkly Rep 2017; 66(23): 615–21. 10.15585/mmwr.mm6623e1 28617773 PMC5657842

[12] Hoen B, Schaub B, Funk AL, Ardillon V, Boullard M, Cabié A, et al. Pregnancy Outcomes after ZIKV Infection in French Territories in the Americas. N Engl J Med 2018; 378(11): 985–94. 10.1056/NEJMoa1709481 29539287

[13] Pomar L, Vouga M, Lambert V, Pomar C, Hcini N, Jolivet A et al. Maternal-fetal transmission and adverse perinatal outcomes in pregnant women infected with Zika virus: prospective cohort study in French Guiana. BMJ 2018; 363:k4431 10.1136/bmj.k4431 30381296 PMC6207920

[14] Pomar L, Malinger G, Benoist G, Carles G, Ville Y, Rousset D, et al. Association between Zika virus and fetopathy: a prospective cohort study in French Guiana. Ultrasound Obstet Gynecol 2017; 49(6): 729–36. 10.1002/uog.17404 28078779

[15] Adhikari EH, Nelson DB, Johnson KA, Jacobs S, Rogers VL, Roberts SW, et al. Infant outcomes among women with Zika virus infection during pregnancy: results of a large prenatal Zika screening program. Am J Obstet Gynecol 2017; 216(3): 292.e1–292.e8. 10.1016/j.ajog.2017.01.018 28153665

[16] Villar J, Papageorghiou AT, Pang R, Ohuma EO, Ismail LC, Barros FC, et al. The likeness of fetal growth and newborn size across non-isolated populations in the INTERGROWTH-21^st^ Project: the Fetal Growth Longitudinal Study and Newborn Cross-Sectional Study. Lancet Diabetes Endocrinol 2014; 2(10): 781–92. 10.1016/S2213-8587(14)70121-4 25009082

[17] Rice ME, Galang RR, Roth NM, Ellington SR, Moore CA, Valencia-Prado M, et al. Vital Signs: Zika-Associated Birth Defects and Neurodevelopmental Abnormalities Possibly Associated with Congenital Zika Virus Infection—U.S. Territories and Freely Associated States, 2018. MMWR Morb Mortal Wkly Rep. 2018;67(31):858–67. 10.15585/mmwr.mm6731e1 30091967 PMC6089332

[18] Delaney A, Mai C, Smoots A, Cragan J, Ellington S, Langlois P, et al. Population-Based Surveillance of Birth Defects Potentially Related to Zika Virus Infection—15 States and U.S. Territories, 2016. MMWR Morb Mortal Wkly Rep. 2018;67(3):91–6. 10.15585/mmwr.mm6703a2 29370151 PMC5812309

[19] Orioli IM, Dolk H, Lopez-Camelo JS, Mattos D, Poletta FA, Dutra MG, et al. Prevalence and clinical profile of microcephaly in South America pre-Zika, 2005–14: prevalence and case-control study. BMJ 2017; 359: j5018. 10.1136/bmj.j5018 29162597 PMC5696624

[20] Morris JK, Rankin J, Garne E, Loane M, Greenlees R, Addor MC, et al. Prevalence of microcephaly in Europe: population based study. BMJ 2016; 354: i4721. 10.1136/bmj.i4721 27623840 PMC5021822

[21] Pasquier C, Joguet G, Mengelle C, Chapuy-Regaud S, Pavili L, Prisant N, et al. Kinetics of anti-ZIKV antibodies after Zika infection using two commercial enzyme-linked immunoassays. Diagn Microbiol Infect Dis 2018; 90(1): 26–30 10.1016/j.diagmicrobio.2017.09.001 29107414

[22] Barzon L, Percivalle E, Pacenti M, Rovida F, Zavattoni M, Del Bravo P, et al. Virus and Antibody Dynamics in Travelers With Acute Zika Virus Infection. Clin Infect Dis 2018; 66(8): 1173–80. 10.1093/cid/cix967 29300893

[23] Matheus S, Tall C, Labeau B, de Laval F, Briolant S, Berthelot L, et al., Performance of 2 Commercial Serologic Tests for Diagnosing Zika Virus Infection. Emerg Infect Dis 2019; 25 (6): 1153–1160 10.3201/eid2506.180361 31107211 PMC6537740

[24] Hoen B, Carpentier M, Gaete S, Tressières B, Herrmann-Storck C, Vingadassalom I, et al. Kinetics of anti-Zika virus antibodies after acute infection in pregnant women. *J Clin Microbiol* 2019; 57(11):e01151–19. 10.1128/JCM.01151-19 31462546 PMC6813021

[25] Euroimmun. Euroimmun test systems for the diagnosis of Zika virus infections. Available online at: https://www.euroimmun.com/documents/Indications/Infections/Zika-virus/HI_2668_I_UK_B.pdf

[26] Dommergues M, Mandelbrot L, Mahieu Caputo D, Boudjema N, Durand Zaleski I, ICI Group Club de médecine foetale. Termination of pregnancy following prenatal diagnosis in France: how severe are the foetal anomalies? Prenat Diagn. 2010; 30(6): 531–9. 10.1002/pd.2510 20509152

[27] Garne E, Khoshnood B, Loane M, Boyd PA, Dolk H, EUROCAT Working Group. Termination of pregnancy for fetal anomaly after 23 weeks of gestation: a European register-based study. BJOG: An International Journal of Obstetrics & Gynaecology. 2010;117(6):660–6. 20374608 10.1111/j.1471-0528.2010.02531.x

[28] Pan American Health Organization / World Health Organization. French Guiana, Guadeloupe, and Martinique. Health in the Americas 2007: Volume II–Countries: 344–360. Available online at: https://www.paho.org/hq/dmdocuments/2010/Health_in_the_Americas_2007-French_Guiana_Guadeloupe_and_Martinique.pdf

[29] Bérard A, Abbas-Chorfa F, Kassai B, Vial T, Nguyen KA, Sheehy O, Schott AM. The French Pregnancy Cohort: Medication use during pregnancy in the French population. PloS One. 2019; 14(7):e0219095. 10.1371/journal.pone.0219095 31314794 PMC6636733

[30] Carvalho MS, Freitas LP, Cruz OG, Brasil P, Bastos LS. Association of past dengue fever epidemics with the risk of Zika microcephaly at the population level in Brazil. Scientific reports. 2020;10(1):1–9. 10.1038/s41598-019-56847-4 32019953 PMC7000767

[31] Campos MC, Dombrowski JG, Phelan J, Marinho CR, Hibberd M, Clark TG, Campino S. Zika might not be acting alone: Using an ecological study approach to investigate potential co-acting risk factors for an unusual pattern of microcephaly in Brazil. PLoS One. 2018; 13(8):e0201452. 10.1371/journal.pone.0201452 30110370 PMC6093667

[32] de Souza WV, Vazquez E, Bezerra LC, Mendes AD, Lyra TM, de Araujo TV, et al. Microcephaly epidemic related to the Zika virus and living conditions in Recife, Northeast Brazil. BMC public health. 2018;18(1):130. 10.1186/s12889-018-5039-z 29329574 PMC5767029

[33] Albert PS, Grantz KL. Fetal growth and ethnic variation. Lancet Diabetes Endocrinol 2014; 2(10): 773 10.1016/S2213-8587(14)70186-X 25258202 PMC10428134

[34] Liu S, Metcalfe A, Leon JA, Sauve R, Kramer MS, Joseph KS. Evaluation of the INTERGROWTH-21st project newborn standard for use in Canada. PLoS One 2017; 12(3): e0172910. 10.1371/journal.pone.0172910 28257473 PMC5336248

[35] Cheng YK, Leung TY, Lao TT, Chan YM, Sahota DS. Impact of replacing Chinese ethnicity-specific fetal biometry charts with the INTERGROWTH-21st standard. BJOG 2016; 123(Suppl 3): 48–55 10.1111/1471-0528.14008 27627597

[36] Silva AA, Barbieri MA, Alves MT, Carvalho CA, Batista RF, Ribeiro MR, et al. Prevalence and Risk Factors for Microcephaly at Birth in Brazil in 2010. Pediatrics. 2018;141(2): e20170589 10.1542/peds.2017-0589 29305391

[37] Ades AE, Soriano-Arandes A, Alarcon A, Bonfante F, Thorne C, Peckham CS, Giaquinto C. Vertical transmission of Zika virus and its outcomes: a Bayesian synthesis of prospective studies. Lancet Infect Dis. S1473-3099(20)30432-1 10.1016/S1473-3099(20)30432-1 33068528 PMC7992034

[38] Manicklal S, Emery VC, Lazzarotto T, Boppana SB, Gupta RK. The “silent” global burden of congenital cytomegalovirus. Clin Microbiol Rev 2013; 26(1): 86–102 10.1128/CMR.00062-12 23297260 PMC3553672

[39] De Santis M, Cavaliere AF, Straface G, Caruso A. Rubella infection in pregnancy. Reprod Toxicol 2006; 21(4): 390–8 10.1016/j.reprotox.2005.01.014 16580940

